# [Retracted] Effect of metformin on cell proliferation, apoptosis, migration and invasion in A172 glioma cells and its mechanisms

**DOI:** 10.3892/mmr.2024.13271

**Published:** 2024-06-25

**Authors:** Zhang Sheng Xiong, Song Feng Gong, Wen Si, Taipeng Jiang, Qing Long Li, Tie Jun Wang, Wen Jie Wang, Rui Yue Wu, Kun Jiang

Mol Med Rep 20: 887–894, 2019; DOI: 10.3892/mmr.2019.10369

Following the publication of this paper, it was drawn to the Editors’ attention by a concerned reader that the cell invasion and migration assay data shown in Fig. 6 and the cell proliferation assay experiments shown in Fig. 2 were strikingly similar to data appearing in different form in other articles by different authors; furthermore, in Fig. 2, for the ‘10 mM metformin’ experiment, certain of the glioma cells appeared to be strikingly similar to other cells contained within the same data panels. Owing to the fact that the contentious data in the above article had already been published elsewhere or were under consideration for publication prior to its submission to *Molecular Medicine Reports*, and owing to concerns with the authenticity of certain of the data, the Editor has decided that this paper should be retracted from the Journal. The authors were asked for an explanation to account for these concerns, but the Editorial Office did not receive a reply. The Editor apologizes to the readership for any inconvenience caused.

